# Biosafety and biobanking: Current understanding and knowledge gaps

**DOI:** 10.1016/j.bsheal.2021.06.003

**Published:** 2021-10

**Authors:** Julie Roux, Maissa Zeghidi, Stephanie Villar, Zisis Kozlakidis

**Affiliations:** a World Health Organization, International Agency for Research on Cancer, Lyon, France; b Ecole Supérieure de Biologie-Biochimie-Biotechnologies, Université Catholique de Lyon, Lyon, France

**Keywords:** Biosafety, Biobank, Data safety, Biological safety, Safety regulations

## Abstract

Infectious disease outbreaks, such as 'Coronavirus disease 2019′ (COVID-19), can constitute major global health threats with far-reaching consequences. As outbreaks develop, the international scientific community must provide high-quality scientific research-ready biological samples to solve the existing clinical and epidemiological questions to better combat the pandemic. Such examples are provided by dedicated biobank facilities, the latter collecting increasingly high volumes of biological samples. However, the more significant concentrations of infectious or potentially infectious biological materials can create a safety risk. The current short report describes the first attempt to identify the published scientific works on biobanking and safety. Three broad thematic areas have been identified: the physical security relevant to staff and sample integrity, the data safety aspects, and the governance parameters relating to the previous two. While the current publications reflect a broad alignment with existing standards and best practices in the biobanking field, they also demonstrate an opportunity for further in-depth work on this field in the post-COVID-19 era.

## Highlights

• Increasing numbers of research-ready biological samples and data are collected in biobanks.
• During the pandemic, such samples can be infectious or potentially infectious collected in large volumes.
• The biosafety considerations for biobanks were reviewed in light of the above.
• Three thematic areas were identified: physical safety, data safety, and governance/compliance.
• There is currently a limited depth of active research on the field, likely to increase in the post-COVID-19 era.

## 1 Introduction

Infectious disease outbreaks, such as 'Coronavirus Disease 2019′ (COVID-19), can constitute major global health threats with far-reaching consequences. The COVID-19 pandemic swept the world within a short period, causing heavy damage to global public health security and human health [1], [2], [3]. As outbreaks develop, the international scientific community must provide high-quality scientific research results to solve the existing clinical and epidemiological questions to better combat the pandemic. The following sample types comprise the essential and fundamental biological materials for such research: blood, serum, throat swabs, sputum, tracheal suction fluid or bronchial lavage fluid, urine, feces. They can be collected after appropriate consent from confirmed patients, asymptomatic infected persons, suspected patients, and their close contacts, as well as from dead patients' cadaver tissues and organs.

The above can prove critical in our understanding and research if accompanied by clinical data – and because the associations are often weak, samples can be needed in large quantities. Simultaneously, several laboratory and analytical processes can accommodate the required high-throughput processing of samples. The implication is clear: if more well-characterized, high-quality pieces are available through biobanks, the faster research will advance and impact healthcare delivery. Thus, biobanking becomes a pivotal element to future treatments' success, relied upon to standardize tissue collection for improved scientific quality. Biobanking is widely defined as a collective term that describes how biological samples (bodily fluid or tissue) and associated data are collected, annotated, stored, and redistributed for future research used to improve our understanding of diseases [4].

The handling of biospecimens in healthcare is not limited only to the technical aspect. Indeed, biobanks have to be subjected to strict ethical and legal regulations, especially those handling potentially infectious materials. Thus, biobanks have to act in concordance with specific regulatory frameworks and to develop strict, auditable procedures and controls ensuring the safety of their staff and quality of their samples for the long term [5], [6]. However, the safety implications of concentrating increasingly larger volumes of samples and data within specific facilities, especially of samples that are potentially infectious, as in the case of COVID-19, have been viewed so far in an empirical way as an integral aspect of different studies.

This manuscript is a short report, systematically searching the literature for the published safety aspects of biobanks focusing on the staff's safety and the integrity of the biological materials. It was considered timely based on the ongoing pandemic and the increasing volumes of biological material collected and utilized in research over the last two decades.

## 2 Method

### 2.1 Data sources and literature search strategy

**Fig. 1:**
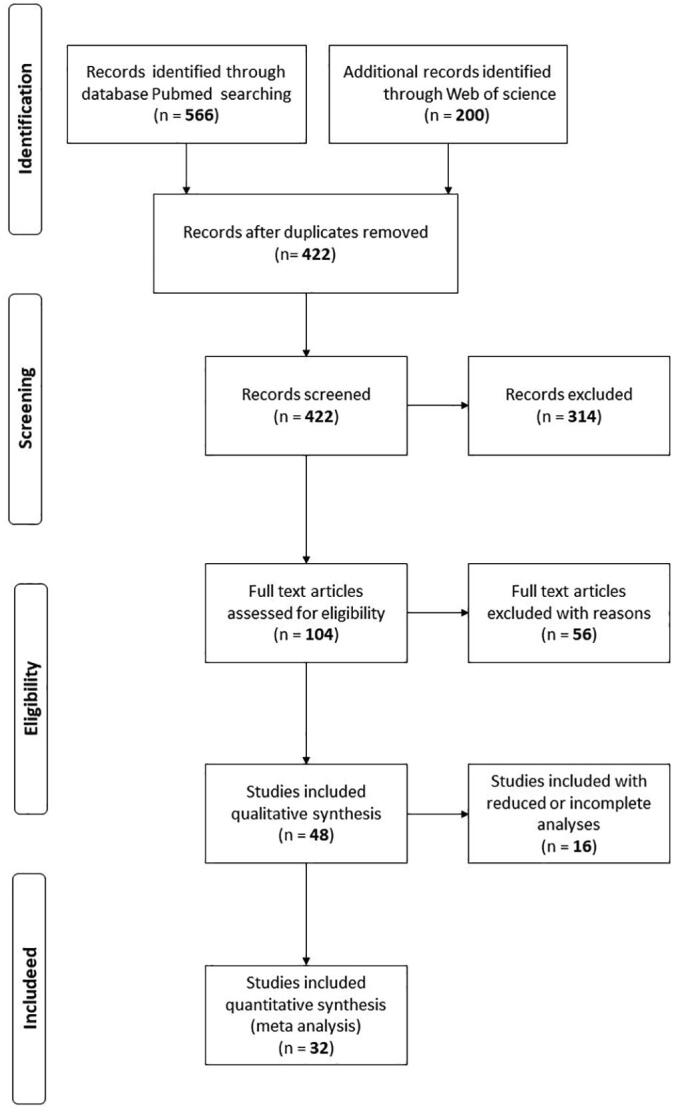
PRISMA graph detailing the search results.

The review of published manuscripts followed the PRISMA guidelines (Fig. 1) [7]. Two investigators (Maissa Zeghidi and Julie Roux) independently conducted a literature search using as combined keywords biobank* or biorepository and safety, security on Pubmed (https://www.ncbi.nlm.nih.gov/pubmed/) and Web of Science (v. 5.35). The database search was run of all the published articles, all languages, from database inception until August 30, 2020. In both databases, the following search strategy was used: pairs of terms were searched as follows: Biobank* AND Safety; Biobank* AND Biosafety; Biorepository AND Safety; Biorepository AND Biosafety; Biobank* AND Security; Biorepository AND Security. Biobank* was used in the search to identify longer forms, such as biobanking. It is thought that these terms would be able to locate the majority of manuscripts within a narrow definition of biosafety and biobanking. However, likely, relevant sections might not be under a separate 'safety' heading and thus more challenging to identify.

### 2.2 Study selection and data synthesis

All studies reporting information on safety, security, and biobanks/biorepositories were included. 766 articles were identified and reviewed independently by two authors (Maissa Zeghidi and Julie Roux), and after all duplicates were removed, 422 articles were considered. After eliminating articles that were not in English, and those that had simply a mention of the words with no further expansion, 104 articles were considered. Of these 104 articles, 56 have a simple definition of the terms and commonly a statement that these aspects constitute necessary parameters, with no analysis or further expansion/exploration of the subject. Hence these articles are included in Supplementary data Table 1 for transparency and other references. However, they were not considered in the current short report. Among the 104 pieces, 32 devoted a considerable amount of the manuscript to expand on those topics, while 16 articles had much reduced or incomplete analyses. These latter categories (48 manuscripts in total, listed in Supplementary data Table 2) were used in the current short report. Any inconsistencies were resolved by consensus with a third author (Zisis Kozlakidis), while thematic groupings (Supplementary data Table 2) and analyses were reviewed by an additional author (Stephanie Villar). All outcomes were included due to the relative scarcity of data.

## 3 Results

The manuscripts identified in this short report (n = 48) followed three loosely defined thematic groups: a) the safe handling of samples and the effects this might have on the well-being of staff (n = 27), b) the safe handling of data related to collected samples (n = 10) and c) the legal and ethical aspects regarding the above aspects (n = 11).

### 3.1 Safe handling of samples and staff safety

The secure handling of biospecimens is one of the first aspects biobanks face. Different steps have to be followed to implement appropriate protocols [8], [9]. For example, one of the first steps often mentioned is to define the type of biobank, then based on this definition, the requirements for establishing a collection including those relating to safety. The guidelines and best practices created through established agencies and biobank networks (e.g., NCI, IARC, ISBER) enable smaller structures to have available information and safely build their system (s). Similarly, biobank networks offer an opportunity to harmonize the existing protocols implemented in the biobanking field [10], [11], [12], in clinical practice [13], as well as in the risk assessment and preparation in the case of natural disasters [14].

Several manuscripts contained more specific examples, where the safety of the collected specimens or the operator has improved through novel protocols [15], [16], [17]. Such technological innovation positively impacts biospecimens' handling and, consequently, on preserving biological indicators [18] or particular cell types [19], [20], [21], [22]. The concept of safety of biological samples extends beyond the immediate collection and handling of biological material and onto the long-term storage conditions. Such aspects were covered in depth using different methodologies, including providing evidence from expert surveys [23] or personal experiences [24], [25], [26]. In other manuscripts, the mention of safety was directly related to the samples' quality [27], [28]. The quality control needs to generate evidence proving the safety of samples during storage. Some quality control protocols were proposed for this purpose [29], [30].

Lastly, the concept of safety within biobanks was linked for some publications to the staff's well-being. The need for informational material that is customized to the operations of the staff members, such as a “newcomer starter pack,” was presented, as well as the need for consistent training on the optimal handling of biospecimens [31], [32], [33], especially in the case of high pathogenicity organisms requiring a P4 laboratory [34].

### 3.2 Safety of data

The second thematic group of manuscripts focused on the safe handling of data related to collected samples. Biobanks need to increasingly consider the concept of data protection according to national and international ethical and legal regulations to guarantee safer management of data [35]. Establishing an appropriate legal framework of operations is often presented as the first step in ensuring data safety. Specifically, in Europe, the General Data Protection Regulation (GDPR) provides a framework with which biobanks need to facilitate information transfer [36]. The in-depth analysis of the GDPR impact and recommendations on its practical implementation is achieved only in a minimal number of manuscripts [37], [38] The general international consensus seems to be that for any transfer of data; three elements are needed: establishing informed consent; the material/data transfer agreements; and a code of conduct [39]. However, imposing such requirements on the safe handling of data necessitates the creation of efficient data infrastructures and databases [40], [41], [42] and integrated methods for the tracking of samples [43], [44].

However, the concept of safe data handling goes beyond GDPR. It includes additional aspects, such as the data's integrity [45], the appropriate handling of data, installing security measures, and staff training to implement the latter effectively. Data security is likely to dominate future discussions on the subject, especially as the number and types of risks increase. For example, data safety risks can vary widely in complexity and impact: malware attacks that compromise clinical and research data; to the Distributed Denial-of-Service (DDoS) attacks, capable of depriving entire institutional systems ability to operate. In particular, cyber-attacks, such as those caused in recent years by repeated Ransomware attacks, have more significant ramifications that go beyond financial loss or privacy breaches [46], [47]. These latter aspects, though necessary, do not appear strongly in the manuscripts identified in the current short report.

### 3.3 Safety is an ethical aspect

The third thematic group of identified manuscripts focuses on the legal and ethical aspects. Ethical and legal regulations permanently evolve in healthcare, reflecting the progress in infrastructure, processing methodologies, or the depth of collected data [48], [49]. The infrastructure-related legal requirements can often be resolved at the technical/engineering level [50]. The legal definitions of consent vary widely between countries, as does the implementation – including examples such as the specific consent process required in Zambia and Tanzania or the more permissive broad consent implemented in Thailand and Nigeria [51]. In much a similar manner, broader ethical challenges can suffer by the lack or misalignment of definitions [52], hence the need for a consistent purpose and application of informed consent [53], [54], even though the latter can also be modified [55]. For example, the new version of the ethics guidelines adopted by the Council for International Organizations of Medical Sciences and WMA World Medical Association caused the inevitable update of consent documents [56] and a different nuanced approach for genetic information [57]. These bioethical constraints have to be taken into account to guarantee the patient's safety and personal information. Using the few available examples identified, in the UK, the UK Biobank succeeded in building trust in the population by relying on altruism, following clear and transparent ethical approaches, and utilizing citizenship language [58]. At the opposite end in the U.S.A., Mexican American individuals view that the lack of available medical research and biobanks negatively acts, disincentivizing individuals to donate their research samples [59].

The above thematic areas reflect existing international independent standards and best practices (e.g., ISO 20387(2018): Biotechnology—biobanking—general requirements for biobanking; ISBER Best Practices) [60], [61], as well as the recently launched (ASCP/ISBER) international training qualification in biorepository science examination for biobank technicians [62]. There is an overall thematic alignment though the depth of research is relatively limited per thematic section.

This publication has some inherent limitations, as it did not consider manuscripts from languages other than English. While much research is published globally in English, safety regulations can often be viewed as a national or sectional competency and become available in the local language. Regarding the selection of manuscripts, only the Pubmed and Web of Science platforms have been used, and none of the pre-print deposition archives. The authors considered this acceptable as there is a relative scarcity of data available on the subject. Hence, the inclusion of pre-print depositions would have been somewhat limited to additional sources and incomplete due to the lack of peer review. Lastly, there may be different manuscripts within national and/or international organizations on the topic that are not accessible through the current search but require individual organizations' queries. Accordingly, these have not been included as they are familiar with many scientific literature reviews.

## 4 Conclusion

The current manuscript demonstrates some dedicated publications already existing, investigating the relationship between biobanking and safety. According to those, the notion of safety can be viewed through three parameters: biological safety (for the individuals handling samples, as well as for the quality and integrity of the biological samples themselves); data safety, for the sample-associated data; and aspects relating to the governance, rules, and regulations. These findings are in line with expectations in terms of developments in the field. However, the overall volume of research (and consequently the number of publications) remains somewhat limited. It is envisaged that in the post-COVID-19 era, these aspects will be reviewed and perhaps even prioritized, highlighting an essential element in the management of samples.

An exciting observation remains that most publications do not feature scientific journals dedicated to biobanking and/or biosafety. On the contrary, they seem to emerge as ad hoc, ancillary parts of existing projects or activities. This ad hoc nature of most publications raises some questions on the overall depth of awareness of individuals working with and biobanks on those aspects. It does not seem to be an aspect of intense research activity. Perhaps the existence of national and international guidelines is considered as sufficiently addressing this point. Furthermore, such elements are likely embedded within the existing protocol and research activities and not necessarily under a separate 'safety' heading. Therefore, identifying such relevant yet less visible sections remains challenging and might require search strategies utilizing additional keywords.

The current short report provides the first evidence on the scientific publications relating to biobanking and safety. The views identified here reflect existing international standards, best practices, and opinions. However, they are not systematically investigated to a greater depth, which creates an opportunity for further work in this field in the post-COVID-19 era.
